# Non-metastatic Non-melanoma Skin Cancers: Our 3 Years of Clinical Experiences

**Published:** 2017-09

**Authors:** Elif Sari

**Affiliations:** Department of Plastic, Reconstructive and Aesthetic Surgery, Faculty of Medicine, Kirikkale University Kirkkale, Turkey

**Keywords:** Nonmelanoma skin cancer, Basal cell carcinoma, Squamous cell carcinoma, Skin cancer

## Abstract

**BACKGROUND:**

Nonmelanoma skin cancers (NMSC) constitute the largest group of skin cancers. In this study, NMSCs were analyzed retrospectively.

**METHODS:**

Between June 2013 and March 2017, demographics and comorbidities of patients underwent reconstructive surgery for NMSC; their risk factors, types, diameters, differentiation, localizations, follow-up times, treatment methods and complications were compared and statistically analyzed.

**RESULTS:**

Totally, 163 tumors [111 basal cell carcinoma and 52 cutaneous squamous cell carcinoma (cSCC)] were excized from 148 patients (63 females, 85 males). Mean age was 70.8 years. Fitzpatrick skin types were between 2-4 and 74 patients. Comorbidities were detected in 63 patients. Tumors were mostly localized in head and neck regions. Forty two lesions in cSCC group were good and 10 were medium differentiated. Defects were reconstructed with flaps in 108 patients. Others underwent primer suturation and grafting. Mean follow-up time was 16.2 months. There was not any complication except one graft failure. There were not significant statistical differences between two groups in terms of skin type, comorbidity, tumor size, fallow-up time and gender values. Conversely, differences of risk factor and age values between the groups were significant. There was a positive correlation between the tumor diameter and poor differentiation in cSCC group. Moreover, there was a negative correlation between tumor size and skin type values in groups.

**CONCLUSİON:**

Our results are quite different from literature needing further multicentric studies on NMSC to clarify the difference.

## INTRODUCTION

Nonmelanoma skin cancers (NMSCs) constitute the largest group of skin cancers and plastic surgeons are the first line in resection and reconstruction of these pathologies. NMSCs include basal cell carcinoma (BCC), cutaneous squamous cell carcinoma (cSCC) and Bowen’ s desease. They are higher than malignant melanoma and cause a large economic loss.^1^^-^^3^ The risk factors for NMSCs increase with age, male gender (men>women), ultraviolet light exposure, fair skin, ionizing radiation, immunosupression, previous skin cancer or premalignant lesions such as actinic keratoses.^4^

BCC is the most frequent malignancy of the skin.^3^^,^^5^ It is usually located on the nose and could be diagnosed by incisional biopsy. The treatment of the lesion could be by nonexcisional and excisional methods which varies according to the risk factors of the tumor.^4^ cSCC is the second most common malignancy of the skin.^6^ Regional lymph node metastasis is the main difference of this tumor from BCC. These lesions could be caused from chronic ulcers or burn wounds.^7^ Definite diagnosis is dependent on the pathological report and the treatment of the lesion should be determined according to the level of the tumor.^4^

Reconstruction choices of these cutaneous malignancies are dependent on multiple factors such as type of the tumor, risk factors of the lesion,^8^ and general health status of the patient. Moreover, the reconstruction ladder should not be forgotten in case of insufficient excision or recurrence of the tumor. We reviewed our 3 years series of 148 consecutive patients with NMSCs and reported the demographic features, risk factors, reconstruction choices, long term results as well as comorbities of the patients. We hope that this limited report could be a pioneer to a multicentric study that contains the features of NMSCs in our country. 

## MATERIALS AND METHODS

A total of 148 patients admitted to our clinic between June 2013-October 2015 and April 2016-March 2017 who underwent reconstructive surgery for NMSC were enrolled. All of the patients and their first degree relatives were informed before the surgery and its potential complications. A detailed information form and photo release form was signed by the individuals or their first degree relatives. Anamnesis and physical examination were recorded and the diagnosis was done with incisional biopsy. All patients with cSCC or basosquamous BCC were underwent ultrasonography to eveluate the possible lymph node metastases. If the lesion had perineural involvement, imaging was done to determine the extent of primary NMSC. 

Patients were prepaired for general anesthesia and the tumors were resected with free margins according to the high risk criterion of the tumors.^8^ Smaller excision margins were obtained in sites where excision and reconstructive options were limited. Reconstruction protocols varied due to the type and localization of the tumor, age and comorbities of the patient and as well as patient’s request. Patients were discharged from the hospital at the day of the operation or the next day. Information were collected retrospectively. The patients who were less than 18 years old, were operated at another center before, followed-up less than 6 months and who had tumors that were extended to the underlying brain, regional lymph node, skull base or distant organ were excluded from the study.

Descriptive and statistical analysis was performed by using SPSS software (Version 17.0, Chicago, IL, USA) and results were compared. Gender, comorbidity, tumor size, follow-up time, Fitzpatrick skin type, risk factors, differantiation of the lesion values that were not normally distributed (p<0.05) and variations that were not homogenous among the groups were included. The results were statistically analyzed by Mann-Whithney U test and *p *values less than 0.05 were considered to be statistically significant. The correlation between tumor diameter and differantiation was analyzed by Pearson Correlation test. Also, the correlation between Fitzpatrick skin type and tumor diameter was analyzed by Pearson Correlation test. As age values were normally distributed (p>0.05), and their variations were homogenous among the groups, therefore, the two independent samples T test was performed for age values and the p values of less than 0.05 were considered to be significant.

## RESULTS

A total of 148 patients with 163 tumors were reconstructed in our clinic. A hundred and eleven of the tumors were BCC and 52 were cSCC (BCC/cSCC ratio=2.1). Forty-seven of all BCC patients were female, 52 of them were male (female/male ratio=0.9) and 16 of all cSCC patients were female, 33 of them were male (female/male ratio=0.48) (Table 1). There was no statistically significant difference in terms of gender between the groups (*p*=0.08) (Table 2).

**Table 1:** Clinical features of the patients and tumors (BCC: Basal cell carcinoma, cSCC: Cutaneous squamous cell carcinoma, F: Female, M: Male)

**Variable**		**BCC**	**cSCC**	**Total**
Gender	F	47	16	63
	M	52	33	85
Age (year)	Mean	67,9	73,7	
Fitzpatrick skin type	Type 2	27	15	42
	Type 3	64	33	97
	Type 4	8	1	9
Comorbidity		38	25	63
Risk factor	None	59	15	74
	Sun exposure (+smoking)	36	24 (8)	68
	Family history	4	0	4
	Immunesupression	0	1	1
	Chronic wound	0	1	1
Tumor localization	Head and neck	105	46	151
	Trunk	3	2	5
	Upper extremity	3	4	7
Tumor diameter (mm)	Minimum	3	3	
	Maximum	45	35	
	Mean	13.4	11.9	
Type of operation	Primer suturation	27	10	37
	Skin graft	9	9	18
	Flap	75	33	108
Follow-up time (months)	Mean	17.2	15.2	
Differentiation	Good	0	42	
	Medium	0	10	
	Bad	0	0	

**Table 2:** Comparisons of skin type, comorbidity, tumor diameter, follow-up time, risk factor and gender values between BCC and cSCC groups. Mann Whithney U Test Results (*p*<0.05 considered to be significant

**Variable**	**Z **	***p*** ** value**
Fitzpatrick skin type	-0.884	0.376
Comorbidity	-1.458	0.145
Tumor diameter	-0.623	0.533
Follow-up time	-1.814	0.07
Risk factor	-3.307	0.001
Gender	-1.71	0.087

Mean age of BCC group was 67.9 years, and it was 73.7 years in cSCC group. There was statistically significant difference between two groups in terms of age values (*p*=0.017). When the risk factors were analyzed between type of the tumors, 59 of all BCC patients did not have any risk factors (60%). Thirty-six subjects reported sun exposure history (36%) and 4 patient had family history for NMSC (4%). Fifteen of cSCC patients had no risk factors (31%) and 24 had experienced sun exposure (65%), 1 patient had immune supression (2%) and another one was with d chronic wound history (2%) (Table 1). 

Eight patients in cSCC group had a history of previous smoking and sun exposure. These patients’ lesions were on their lower lips. Risk factors were significantly different between the BCC and cSCC groups (*p*=0.001) (Table 2). Patients of cSCC group had more risk factors than the BCC group patients. Fitzpatrick skin type was 2 in 27 patients in BCC group and 15 patients in cSCC group. It was 3 in 64 patients in BCC group and 33 patients in cSCC group and it was 4 in 8 patients in BCC group and 1 patient in cSCC group (Table 1). 

There was a negative correlation between tumor diameter and Fitzpatrick skin type values (r=-0.093). A total of 99 patients were diagnosed as BCC, while 61 did not have any comorbidities (61.6%), 38 of them had comorbidities such as hypertension, coronary heart desease, asthma and diabetes mellitus (38.4%). Twenty-five of 49 cSCC patients had comorbidities (51%) and the others did not have any comorbidities (49%) (Table 1). Comorbidity values were not statistically different between BCC and cSCC group (*p*= 0.145) (Table 2). 

A total of 111 BCC lesions were excised from 99 patients. The diameter of the tumor ranged 3-45 mm (mean=13.4 mm). Three of the BCC lesions were diagnosed as basosquamous variation. None of them had perineural invasion or lymphadenopathy. A total of 52 cSCC lesions were reconstructed in 49 patients. Tumors’ diameter were varying from 3 to 35 mm (mean=11.9 mm). Forty-two of them were good differentiated and 10 of them were medium differentiated (Table 1). Tumor diameter values were not significantly different among the groups (*p*=0.533). None of them had lyphadenopathy (Table 2).

Moreover, there was a strong positive correlation between poor tumor differantiation and tumor diameter (r=0.283). A total of 105 tumors in BCC group and 46 tumors in cSCC group were located in head and neck region. Second localization was upper extremity and the last one was trunk. The most common anatomic localizations of the tumors were the nose in BCC group and the cheek in cSCC group (Table 1 and 3). Ten patients had multiple tumors. Surgical treatment was designed according to the rules of the reconstruction ladder. A total of 27 defect was sutured primer in BCC group.

**Table 3:** Localizations of the tumors according to the tumor type (BCC: Basal cell carcinoma, cSCC: Cutanous squamous cell carcinoma)

**Tumor localization**			**BCC**	**cSCC**	**Total**	**Percent**	** Total (%)**
Face	Frontal region		9	3	12	7.3	
	Glabella		1	0	1	0.6	
	Nose		31	4	35	21.4	
	Cheek		24	15	39	24	
	Ear		6	2	8	4.9	
	Eyebrow		3	3	6	3.6	
	Lateral canthus		1	0	1	0.6	79
	Medial canthus		2	0	2	1.1	
	Lower eyelid		4	0	4	2.4	
	Lower lip		1	10	11	6.7	
	Nasolabial sulcus		6	2	8	4.9	
	Upper lip		1	0	1	0.6	
Scalp			14	6	20	12.2	12.2
Neck			2	1	3	1.7	1.7
Trunk			3	2	5	3	3
Upper extremity			3	4	7	4.1	4.1
Total			111	52	163	100	100

**Fig. 1:**
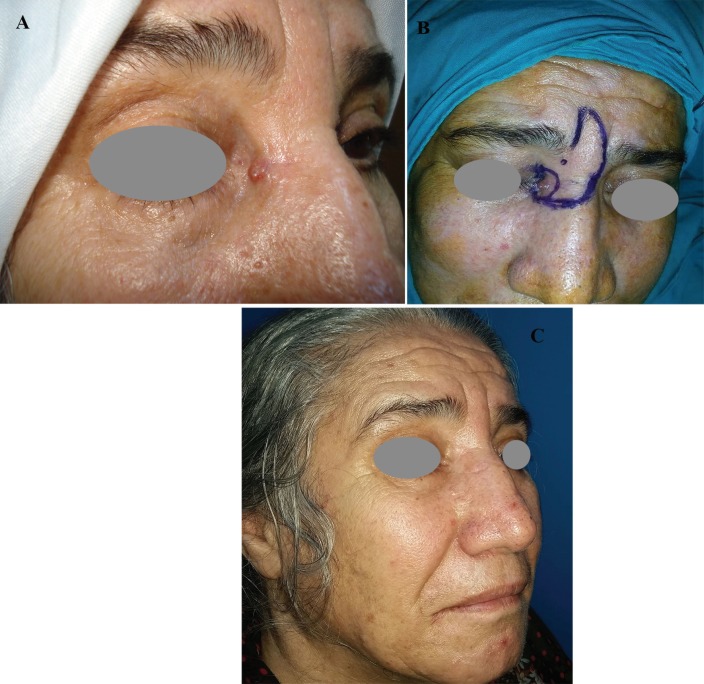
Sixty-eight years old female patient with BCC on her right medial canthus admitted to our clinic. The defect was reconstructed with procerus flap. (a) Preoperative, (b) Intraoperative, and (c) Postoperative 24 months photos of the patient were presented

**Fig. 2:**
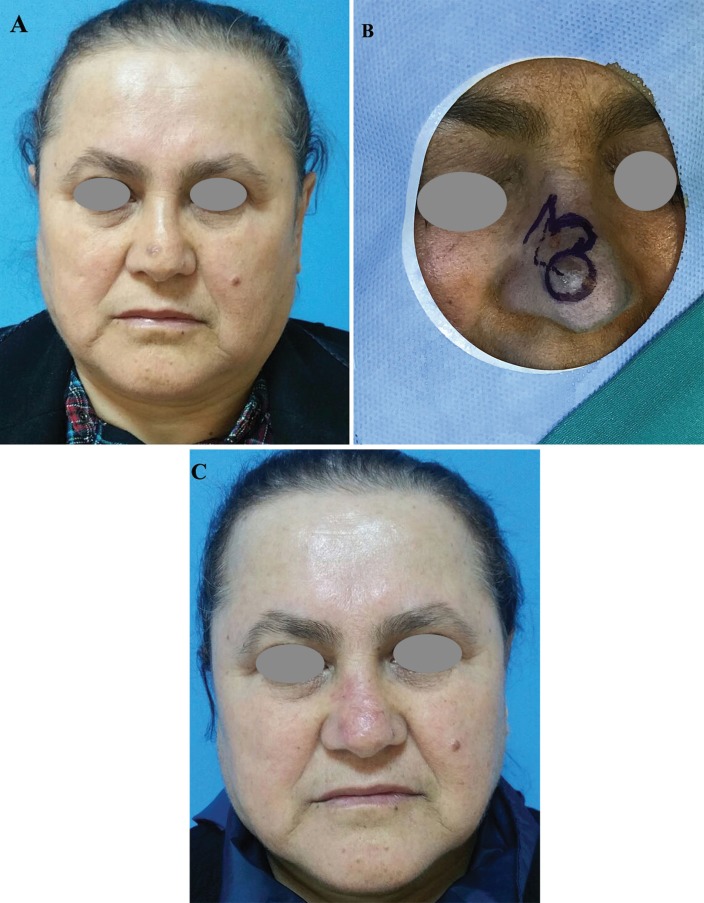
Fifty-four years old female patient with BCC on her nose admitted to our clinic. The defect was covered with bilobed flap. (a) Preoperative, (b) Intraoperative, and (c) Postoperative 24 months photos of the patient were presented

**Fig. 3:**
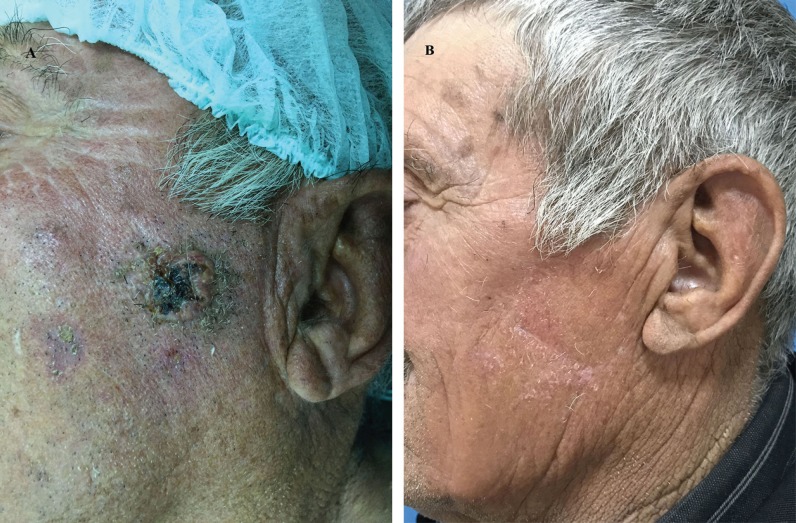
Eighty-nine years old male patient with cSCC on his left cheek was referred to our clinic. The defect was reconstructed with rhomboid flap. (a) Preoperative, and (b) Postoperative 12 months photos of the patient were presented

**Fig. 4:**
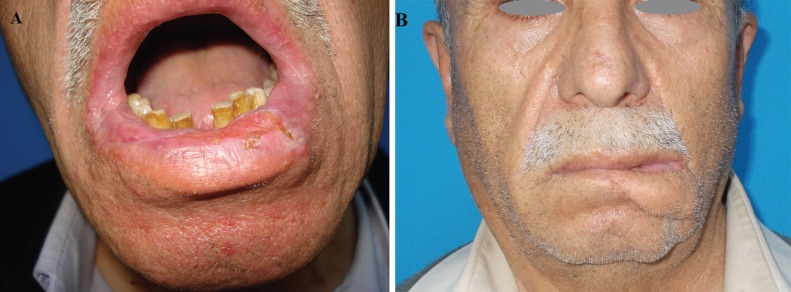
Sixty-two years old male patient with cSCC on his lower lip was admitted our clinic. The defect was reconstructed with left Fujimori Gate flap. (a) Preoperative, and (b) Postoperative 12 months photos of the patient were presented

Skin graft was adapted in 9 patients. Seventy-five flaps were elevated in BCC patients. The mean fallow-up time was 17.2 months in BCC group (Table 1). Only 1 skin graft failured in a patient. Debridement was done and a skin flap was advanced from the nasolabial sulcus. No further complications developed in the reconstruction procedures of BCC group (Figure 1 and 2). A total of 10 defects were closed with primary suturing in cSCC group. Nine of the cSCC patients were reconstructed with skin graft and the remaining patients underwent flap surgery. The mean fallow-up period of the patients was 15.2 months (Table 1). There was no complication in cSCC patients (Figure 3 and 4). 

## DISCUSSION

Number of patients diagnosed as skin cancer doubled in last 4 years and these pathologies are an important health problem in our country.^9^ They are more common in men and age is an important risk factor for development of these tumors. We could not find any detailed information about NMSC in our country due to inconsistent data collection. However, BCC/cSCC ratio was 2.1 according to the present study. This result was lower than in the literature that the BCC/cSCC ratio is 3.^10^ Moreover, Karadeniz *et al.* reported the ratio as 1.1^11^ and Ülkür *et al.* found it similar with the literature in their study.^12^ Therefore, we think that BCC/cSCC ratio could be different from literature in all around of our country. However, further multicentric studies should be done to evaluate the correct ratios.

NMSC are usually seen in older ages (> 40 years). In our study, the mean age of our patients was 70.4 years (Range=39-97 years). The mean age was 67.6 in BCC, 77.3 in basosquamous variant and 73.7 in cSCC. Moreover, age of the patients of cSCC group was higher than BCC group and the difference was significant. Therefore, cSCC occurred at older ages in comparison to BCC. BCC was previously reported equal in men and women^6^^,^^13^^,^^14^ and female/male ratio of cSCC was shown to be 1/2 in the literature.^15^^,^^16^

In our study, this ratio was 0.9 in BCC group and 0.48 in cSCC group which was similar with the literature. However, there was not significant differences between the BCC and cSCC groups in terms of gender values. Therefore, there was no significant gender dominance between these two types of tumors. Several risk factors could be mentioned about NMSCs. The major one is chronic sun exposure among them. Arsenic and repeated contact with polycyclic hydrocarbons, immunesupression, family history, cronic wounds and burns are the other risk factors for these tumors^.^^7^

In our study, 59 patients in BCC group (59.5%) and 15 patients in cSCC group (30.6%) did not have any risk factor. Thirty-six patients in BCC group and 32 patients in cSCC group had chronic sun exposure according to our data. Eight of these 32 patients were smokers and these patients’ lesions were on their lower lip. These information support the literature. The other risk factors were family history in 4 patients in BCC group, immune supression in 1 patient and chronic wounds in 1 patient in cSCC group. Risk factors were significantly different between the BCC and cSCC groups. Patients of cSCC group had more risk factors than the BCC group patients. 

Skin type is the other factor about NMSC development. Fair skin type (Fitzpatrick 1 and 2) is more prone to skin cancer formation. In the present study, 27 patients in BCC group and 15 patients in cSCC group had Fitzpatrick type 2, 64 patients in BCC group and 33 patients in cSCC group had Fitzpatrick type 3 and 8 patients in BCC group and 1 patient in cSCC group had Fitzpatrick type 4 skin features. Therefore, our data conflicts with the literature. On the contrary, there was a negative correlation between skin type value and tumor diameter according to our study, while the skin type value increased, the diameter of the tumor decreased. Therefore, we could report that skin type is more associated with tumor size than tumor development.

NMSCs are usually settled in the head and neck region.^15^ In our study, 92% of our patients had NMSC in their head and neck. Nose was the most affected area in head and neck region (21.4%) and upper extremity was the second settlement area (4.1%). However, trunk is the second anatomic localization according to another study.^12^ Multiple tumor ratio was 47% in the literature,^17^ but only 10 patients (6.7%) have multiple tumors in our data. Our multiple tumor ratio is similar with the study of Karadeniz *et al.*^11^ So multiple NMSC patient ratio could be lower in our country than the literature. 

Treatment of the skin cancers could be classified as nonsurgical and surgical methods. Surgical treatment of the tumor was performed according to several factors such as risk factors of the tumors, diameter of the tumor, anatomic localization of the lesion and also comorbidities of the patient. Our patients were treated with surgical methods such as primary suturation, and skin grafts and flaps. Reconstruction ladder rules were performed in most patients. However, in our participants, important aesthetic areas such as face was usually reconstructed with flap surgery instead of skin grafting. Similarly, flap surgery was performed more often than other surgical options in another study.^18^

Finally, skin cancers are preventable and treatable pathologies with sun protection and early diagnosis. The present study gives the informations about a small population’s NMSC statistics. We think that we need to have better knowledge about the distribution of skin cancers in our country by the necessary notifications about the NMSC and multicentric studies.
